# Pregnancy-Associated Atypical Hemolytic Uremic Syndrome and Life-Long Kidney Failure

**DOI:** 10.7759/cureus.25655

**Published:** 2022-06-04

**Authors:** Mohsin Mirza, Nazia Sadiq, Chawmay Aye

**Affiliations:** 1 Internal Medicine, Woodhull Medical Center, Brooklyn, USA; 2 Nephrology, Woodhull Medical and Mental Health Center, Brooklyn, USA

**Keywords:** eculizumab, thrombotic micro-angiopathy, renal failure, pregnancy, dialysis, atypical hemolytic uremic syndrome

## Abstract

Atypical hemolytic uremic syndrome (aHUS) is a rare disorder characterized by a triad of thrombocytopenia, thrombotic microangiopathy, and acute renal failure. The background pathogenesis of aHUS stems from mutations in the genes of the complement cascade. However, certain circumstances including normal physiological conditions such as pregnancy, environmental factors, or triggers, can activate genetically predisposed individuals and lead to aHUS. We present a case of a young female who presented with acute renal failure and later was diagnosed with aHUS. Possible potential triggers were investigated, and it is believed that pregnancy was associated with the development of aHUS in this young genetically predisposed female leading to life-long acute renal failure. This case highlights a unique case of a devastating systemic disease triggered by a normal physiological phenomenon. Moreover, it reiterates the importance of early diagnosis and how it is imperative to proceed to treatment promptly to prevent chronic renal failure. Further reporting of cases is warranted to monitor the incidence and improve prognostic outcomes in patients with aHUS.

## Introduction

Atypical hemolytic uremic syndrome (aHUS) is an extremely rare disorder with an estimated incidence of between 0.5 and 2 patients per year. It is characterized by a triad of thrombocytopenia, thrombotic microangiopathy, and acute renal failure. Although the background pathogenesis of aHUS is associated with mutations in the genes of the regulatory proteins involved in the complement cascade, a genetic mutation by itself is not enough to cause aHUS [1]. Certain circumstances, environmental factors, or triggers can activate genetically predisposed individuals and lead to aHUS. We present a case in which pregnancy triggered the activation of aHUS in a genetically predisposed young female which rapidly led to acute renal failure.

## Case presentation

An 18-year-old female presented to the emergency department with complaints of progressively worsening epistaxis, on and off for one year, involving both nares and associated with the passage of clots. Her past medical history was significant for pre-eclampsia and preterm delivery via C-section at 26-weeks of gestation. The patient was two years post-partum at the time of initial presentation. A review of symptoms was positive for headaches and irregular menstrual cycles. Physical examination was remarkable for blood pressure (BP) of 161/104 mmHg, marked conjunctival pallor with left-sided costovertebral angle tenderness, and a split S2. The rest of the physical examination was unremarkable. 

Initial laboratory investigation revealed hemoglobin of 7.2 g/dl, hematocrit of 22.7%, and platelet count of 163000/μL. Chemistry was remarkable for a blood-urea-nitrogen (BUN) of 120 mg/dL, creatinine of 17.47 mg/dl (baseline creatinine two years ago during pregnancy was 1.1), glomerular filtration rate of 3.2 mL/min, and phosphorus of 8.7 mg/dL. Coagulation parameters (activated partial thromboplastin time (aPTT), prothrombin time (PT)/international normalized ratio (INR)), reticulocyte count, haptoglobin, lactate dehydrogenase (LDH), and aspartate aminotransferase (AST)/alanine aminotransferase (ALT) were within normal ranges. Urine analysis was significant for 300 mg/dl protein, moderate blood, and trace ketones. Spot protein was 4550 mg/gram roughly equivalent to 4.6 g/day of proteinuria. Cardiac markers and urine toxicology screen were negative. EKG showed sinus rhythm, and chest X-ray revealed no acute pathology. The patient was admitted with the impression of hypertensive emergency in the setting of acute renal failure. 

**Figure 1:**
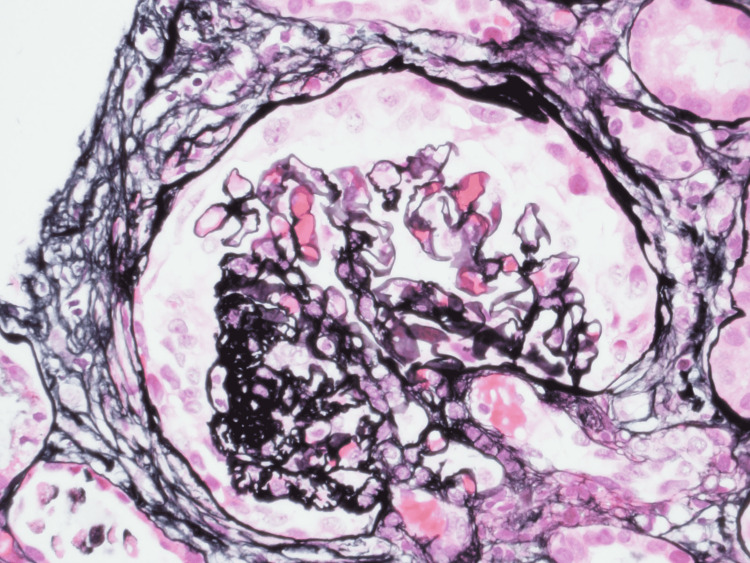
Light microscopic image of glomeruli with collapsed capillary tuft

**Figure 2:**
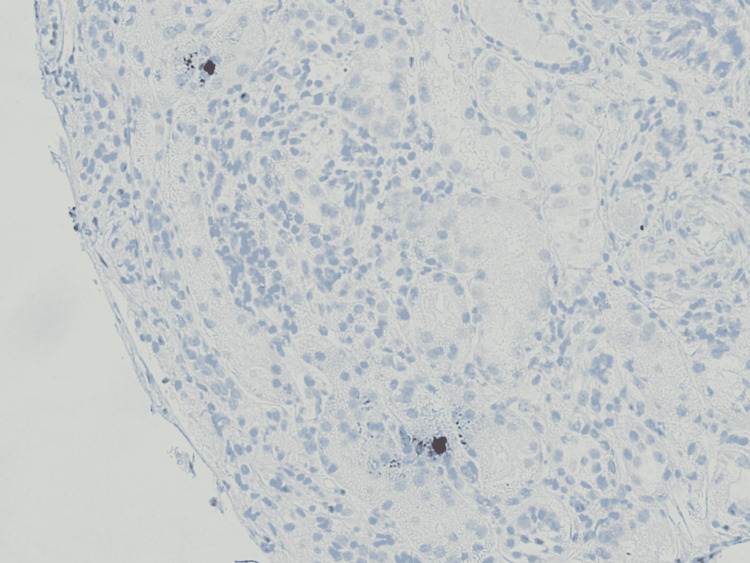
Light microscopic image showing tubular atrophy, interstitial fibrosis, and scant inflammatory cells

On the medical floor, the patient’s BP was stabilized, and nephrology was consulted for evaluation of acute renal failure. Further laboratory workup was negative for autoimmune and vasculitis etiology, i.e., anti-DNA, anti-smooth muscle antibody (ASMA) AB, c-antineutrophil cytoplasmic antibodies (ANCA), p-ANCA, antiphospholipid AB, lupus anticoagulant; HIV and hepatitis serologies were negative. No initial peripheral smear was present. C3 was mildly decreased at 66 mg/dL (ref range 81-157 mg/dL), with normal C4 levels. Kidney ultrasound showed increased renal cortical echogenicity. Considering the acuity of renal failure, the patient underwent renal biopsy which showed thrombotic microangiopathy, global and focal segmental glomerulosclerosis with collapsing features, low grade immune complex mediated glomerulonephritis, severe arterio-nephrosclerosis, and 90% of interstitial fibrosis/tubular atrophy (Figures 1-2). Patient was started on hemodialysis during the admission with no further acute complications during the hospital stay. She was discharged with three times weekly maintenance dialysis and follow up with hematology and nephrology for further care. 

Subsequent workup with hematology was remarkable for schistocytes on peripheral smear, persistently low hemoglobin ~10 gm/dl, low platelets ~120 K, elevated reticulocyte count of 2.87% (reference range 0.5%-1.7%), ADAMT13 activity level of 76 (reference range >61) and negative stool culture for Shiga toxin and entero-hemorrhagic Escherichia coli 0157:H7. Atypical HUS was suspected, and patient’s renal biopsy slides were sent for complement membrane attack complex (C5b-9) staining, which returned positive. Further genetic testing was remarkable for negative prothrombin G20210A mutation, negative factor V Leiden mutation, and positive mutation in the CFHR1 gene. A diagnosis of aHUS was made based on clinical history, renal biopsy findings, and genetic testing. She was started on eculizumab injection every two weeks while waiting for possible workup for renal transplant. 

## Discussion

Hemolytic uremic syndrome (HUS) is a type of thrombotic microangiopathy consisting of intravascular hemolysis, thrombocytopenia, and thrombi in small vessels and capillaries, leading to organ damage, most commonly acute renal failure. It is commonly divided into two types based on likely etiology, typical HUS and atypical HUS. 

Typical HUS, caused by Shiga-like toxic producing Escherichia coli (STEC) infection, accounts for 90% of the cases of HUS [2]. The pathogenesis of the remaining 10% of HUS, also known as atypical HUS, stems from genetic mutations in one of the multiple genes (commonly C3, CFB, CFH, CFI, MCP (CD46), and CFH-CFHR) encoding the proteins which are part of the complement pathway [3]. However, as previously discussed, a genetic mutation is not enough to lead to the presentation of aHUS by itself. It only leads an individual to become genetically predisposed to the development of aHUS in the presence of triggers. Common triggers include the presence of an acute infection, H1N1 influence, varicella, underlying cancer, autoimmune disease, drugs, or pregnancy. 

Initial presentation of atypical HUS can vary from vague symptoms of fatigue and illness to dyspnea, bleeding, and high blood pressure. Patients with aHUS are more likely to develop serious, often lifetime complications such as renal failure, requiring hemodialysis, if symptoms are not identified in the early course of the presentation and if treatment is delayed. An observational study in France of 214 patients with aHUS showed that 29% and 56% of children and adults, respectively, progressed to end-stage renal disease (ESRD) or death within a year of follow-up [3]. Diagnosis is complicated and involves a clinical history consistent with hemolytic anemia, thrombocytopenia, and kidney dysfunction in the presence of identified genetic mutations in genes associated with aHUS, or when two or more members of the same family are affected by the disease for at least six months apart. Other more common thrombotic microangiopathies such as thrombotic thrombocytopenic purpura (TTP) and typical hemolytic-uremic syndrome share similar presenting characteristics and hence make it critical for clinicians to include aHUS in their differentials when considering the diagnosis of thrombotic microangiopathies. The absence of a history of diarrhea, negative stool cultures for Shiga toxin, hypertension, hematuria, and proteinuria should prompt the nephrologist towards a probable diagnosis of aHUS and warrant further workup with hematology. Upon diagnosis, treatment with supportive care and immunotherapy should be prompted. 

A trigger from pregnancy accounts for 20% of all aHUS cases reported in women [4]. A systemic review of literature has shown only 60 rare cases of pregnancy-triggered aHUS with 66 pregnancies. Fifty-four out of the 60 pregnancies (~90 percent) were associated with nulliparous women (58%), occurred post-partum (94%), and were more often after cesarean delivery (70%) [4]. Common preceding obstetric complications included pre-eclampsia, hemorrhage, and fetal death. Women with aHUS associated with first-time pregnancy achieved higher rates of disease remission when treated with eculizumab compared to those who did not receive treatment with eculizumab (88% vs 57%, P=.02) [4].

Treatment modalities have included plasma exchange and eculizumab, a monoclonal antibody against complement protein C5. The FDA has approved the drug since 2011 for the management of aHUS [5].

Multiple studies have demonstrated the high effectiveness of eculizumab. A case study in an adolescent with relapsing unclassified aHUS demonstrated the temporary termination of the microangiopathic hemolytic activity upon initiation of eculizumab. However, continued renal damage as a result of the preceding and subsequent aHUS activity ultimately led to ESRD in the same patient, therefore pointing to the strong likelihood that therapeutic success may depend on early initiation of eculizumab. The optimal duration of therapy is variable and remains to be determined [6]. 

Our case represents a similar presentation of a very rare case in which a nulli-gravid female with an obstetric history complicated with pre-eclampsia was found to have acute renal failure secondary to atypical HUS. Her initial delayed presentation (lost to follow-up for two years) led to the delayed diagnosis and lifetime complication of acute renal failure requiring hemodialysis. There remains limited clinical data and treatment experience in pregnant or post-partum females diagnosed with aHUS and the timeline between initial presentation and the onset of aHUS and/or acute renal failure. 

## Conclusions

aHUS remains a rare disorder, with overlapping clinical symptoms to many other similar disease processes, which poses a challenge to clinicians and delays diagnosis. This case highlights a unique case of a devastating systemic disease triggered by a normal physiological phenomenon of pregnancy leading to advanced morbidity. Moreover, it reiterates the importance of early diagnosis and how it is imperative to proceed to treatment promptly to prevent chronic renal failure.
